# 

**Published:** 1903-08-15

**Authors:** 


					﻿THE
DENTAL REGISTER
EDITED BY
NELVILLE S. HOFF, D. D.S.,
ANN ARBOR, MICH.
Vol. LVII. AUGUST 15, 1903.	. | E
PRICE, ONE DOLLAR A YEAR IN ADVANCE.
PUBLISHED MONTHLY BY
SAMUEL A. CROCKER & CO.,
THE OHIO DENTAL AND SURGICAL DEPOT.
3S to 39 \V. t>tli iSt., Cinciiinnti, O.
Entered at the Postoffice at Cincinnati, 0., as second-class mail matter.
				

